# Diversity of Mating-Type Chromosome Structures in the Yeast *Zygosaccharomyces rouxii* Caused by Ectopic Exchanges between *MAT*-Like Loci

**DOI:** 10.1371/journal.pone.0062121

**Published:** 2013-04-16

**Authors:** Jun Watanabe, Kenji Uehara, Yoshinobu Mogi

**Affiliations:** Manufacturing Division, Yamasa Corporation, Araoicho, Choshi, Chiba, Japan; SUNY at Buffalo, United States of America

## Abstract

We investigated sex chromosome diversity in *Zygosaccharomyces rouxii* (*Z. rouxii*). In the current study, we show that the organization of the mating-type (*MAT*) locus is highly variable in the *Z. rouxii* population, indicating the *MAT*, *HML*, and *HMR* loci are translocation hotspots. Although NBRC1130 and CBS732 were originally two stocks of the type strain of the species, only NBRC1130 retains the original karyotype. A reciprocal translocation between the *MAT* and *HMR* loci appears to have occurred during the early passage culture of CBS732, which was used for genome sequencing. In NBRC1733, NBRC0686, NBRC0740 and NBRC1053, the terminal region of the chromosome containing the *HMR* locus was replaced with the chromosomal region to the left of the *MAT* or *HML* loci. The translocation events found in NBRC1733, NBRC0686, NBRC0740, and NBRC1053 were reconstructed under our experimental conditions using the DA2 background, and the reconstruction suggests that the frequency of this type of translocation is approximately 10^−7^. These results suggest that the *MAT* and *MAT*-like loci were the susceptible regions in the genome, and the diversity of mating-type chromosome structures in *Z. rouxii* was caused by ectopic exchanges between *MAT*-like loci.

## Introduction

Chromosomal rearrangements, which include translocations and large-scale deletions, play key roles in phenotypic variation, genome evolution and genetic disease [1], [2]. Multi-copy genes such as retrotransposons are known to elicit these chromosomal rearrangements. During the evolution of the Saccharomycetaceae, translocations have cycled telomere location, and the mating-type (*MAT*) locus has been a deletion hotspot [3], [4].

In model yeast *Saccharomyces cerevisiae*, the *MAT* locus determines sex (mating-type). Two mating type genes (idiomorphs), either *MAT* **a** or *MAT*α, are encoded at the *MAT* locus and enable the locus to specify three cell identity types: haploid **a**, haploid α or diploid **a**/α. The *MAT* locus is often replaced with either *HML*α or *HMR* **a**, which are silenced by chromatin modifications and are therefore called “silent cassettes” [5]. Based on extensive analysis of yeast sex evolution, mating-type switching has evolved from a two-step process: gain of silent cassettes and HO endonuclease [6]. These two-step events occurred after the ancestor of hemiascomycetous yeast had diverged from other families, such as genus *Debaryomyces* and *Kluyveromyces*. Fabre *et al*. have suggested that a remnant of the *HO* gene resides within the *K. lactis* genome [7]. However, Butler showed that this sequence is distantly related to the other *HO* gene and is most similar to a second HO-like sequence present in the *S. castellii* genome [5]. Therefore, the *K. lactis* gene and the *S. castellii* gene likely evolved independently of *HO*, which was acquired late in the evolution of hemiascomycetes [5]. The presence of HO endonuclease enables efficient switching because HO endonuclease catalyzes DNA double-strand breaks (DSBs) at the Y-Z junction of the *MAT* locus and subsequently repairs the break using *HML*α and *HMR* **a** [5]. Chaotic DSBs lead to cell cycle arrest and/or cell death; therefore, *HO* gene expression is strictly regulated by cell type, cell cycle and mother/daughter cell expression. However, whether the *HO* gene was regulated immediately after it was acquired is unknown.

Z. *rouxii* diverged from the *Saccharomyces* lineage after gaining the *HO* gene but before whole-genome duplication (WGD) [6]; hence, chromosomal rearrangements involving mating type conversions may be detected in its genome. The availability of complete genome sequences for hemiascomycetous yeast, including the *Z. rouxii* strain CBS732 [8], provides an ideal opportunity to analyze the consequences of *HO* gene acquisition. A recent study has already demonstrated that switching errors accumulate along evolutionary lineages and have a profound effect on the structure of the *MAT*-containing chromosome in post-WGD species, and that the non-WGD species (except for *Z. rouxii*) retain an organization similar to *Torulaspora delbrueckii*, which has the *MAT* organization *DIC1*-*MAT*-*SLA2* [4]. We wondered why the *MAT* organization of *Z. rouxii* was different from other non-WGD species.

To meticulously characterize yeast sex chromosome evolution, we compared the organization of the *MAT* and *MAT*-like loci in the *Z. rouxii* population. In this paper, we report evidence that sex chromosome diversity abounds in *Z. rouxii*.

## Results

### Isolation of a CBS732-like haploid strain in *Z. rouxii*

Recent studies have shown that various strains originally identified as “*Z. rouxii*” in yeast culture collections comprise at least three groups: *Z. rouxii* type strain group (such as CBS732), *Zygosaccharomyces sp.* group (such as NCYC3042, and also called *Z. pseudorouxii*), and an allopolyploid group (such as ATCC42981 which is a hybrid between the *Z. rouxii* type strain group and *Zygosaccharomyces sp*. group). Therefore, the hybrid strain possesses T and P subgenome derived from CBS732 and NCYC3042, respectively (Fig. S1A) [9], [10]. Solieri *et al*. studied the ploidy level and demonstrated that ATCC42981 and some wild strains isolated from traditional balsamic vinegar had a diploid DNA content [11]. More recently, Solieri *et al*. showed that the group of *Z. rouxii*-related strains is a complex of haploid and diploid heterogeneous species including: 1) the haploid species *Z. rouxii*, e.g., CBS732; 2) the diploid species *Z. sapae*, isolated from high sugar environments; and 3) a diploid mosaic lineage that includes strains retrieved from a salty environment (Fig. S1A) [12], [13]. According to their classification, the allopolyploid species, such as ATCC42981, are categorized as a diploid mosaic lineage.

To remove the diploid species *Z. sapae* and a diploid mosaic lineage from this study, PCR analysis was performed using a T subgenome-specific primer and a P subgenome-specific primer (Table S1). The *ADE2* and *SOD2* alleles encoded by the T subgenome were detected in all strains used in this study except for DA2 [14], an *ade2*-disrupted strain derived from UL4, an *ura3* ^−^ mutant strain derived from CBS732 [15]. In contrast, *ADE2* and *SOD2* encoded by the P subgenome were only detected in the NBRC1876, NBRC1877, and Z3 strains (Fig. S1B). NBRC1876 is allopolyploid [9] or a diploid mosaic lineage [13], and NBRC1877 is a diploid mosaic lineage [13]. Therefore, Z3 could also be allopolyploid or a diploid mosaic lineage strain. The remaining strains (NBRC1130, DL2, DA2, NBRC0686, NBRC0740, NBRC1053, and NBRC1733) do not appear to be hybrids and could be haploid. To further confirm the ploidy status, we performed flow cytometric analysis (Fig. S2). For DA2 and DL2, we obtained two peaks corresponding two subpopulations; one population had 1n content (G0/G1 phases), and the other population had 2n content (G2/M phases) of the haploid genome. A clear shift of the two peaks toward a double amount of DNA was observed for strains NBRC1876, NBRC1877, and Z3. However, no shift of the two peaks was observed for strains NBRC1130, NBRC0686, NBRC0740, and NBRC1733 (Fig. S2). This result also suggests that the NBRC1876, NBRC1877, and Z3 strains are the diploid mosaic lineage, and NBRC1130, NBRC0686, NBRC0740, NBRC1053, and NBRC1733 strains are haploid.

### High variability in the *MAT*-flanking region in *Z. rouxii*

**Figure 1:**
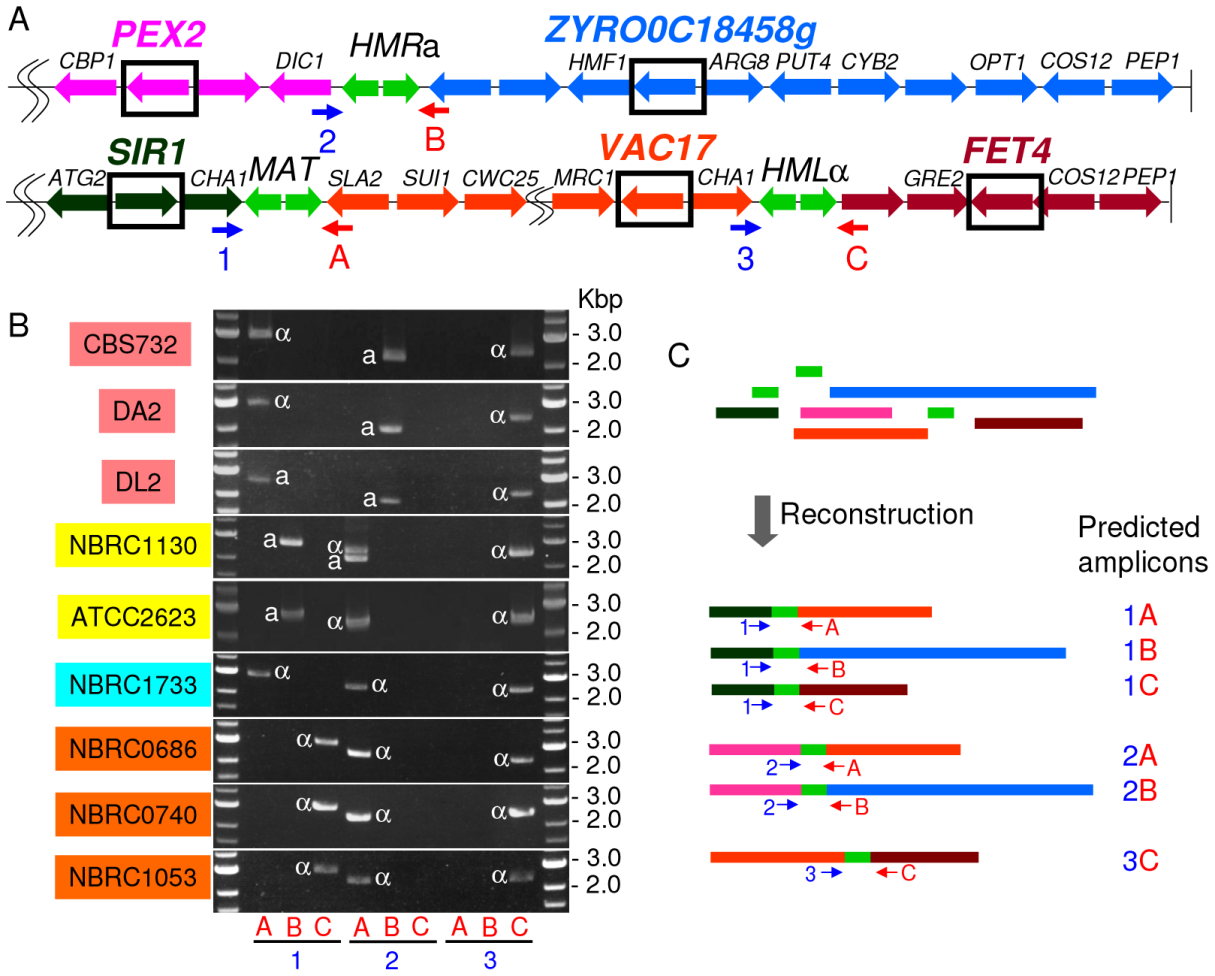
High variability in the *MAT*-flanking region in *Z. rouxii*. (A) Gene organization around the *MAT*-like locus in *Z. rouxii* CBS732, which was used for genomic sequencing. Small arrows indicate primers. The vertical bars are telomeres. The box indicates a gene used for a probe in Fig. 2. (B) PCR amplification of the *MAT*-like locus from *Z. rouxii* CBS732, DA2, DL2, NBRC1130, ATCC2623, NBRC0686, NBRC0740, NBRC1053, and NBRC1733. (C) Arrangement of chromosomes. Partial chromosome structures around the *MAT*-like loci were reconstructed. Theoretically, all possible combinations of pieces to yield PCR amplicons detected in (B) are shown. Additionally, refer to our prediction model in Fig. 4. Coloring corresponds to gene position in CBS732: pink, left side of *HMR*; blue, right side of *HMR*; dark green, left side of *MAT*; orange, between *MAT* and *HML*; and wine red, right side of *HML*.

Gordon *et al*. showed that the genes located immediately adjacent to the *MAT* locus have continually been deleted, truncated, or transposed to other regions in the genome [4]. These deletion and transposition events are caused by evolutionary accidents during mating-type switching, which is facilitated by HO endonuclease. The authors also found that the translocation joined the X side of *MAT* (left side of *MAT* in Fig. 1A) to a telomeric region containing *CHA1* in *Z. rouxii* [4]. If these deletions, truncations, and transpositions are related to the acquisition of HO endonuclease, a remnant of degraded *MAT* organization may be detected in the *Z. rouxii* population. Based on CBS732 genome sequencing data, we carried out PCR analysis by using specific primer sets that annealed to *MAT*, *HML*, and *HMR* flanking regions (outside of the X and Z regions) (Fig. 1A, Table S1). In the CBS732, DA2, and DL2, a PCR product was detected by using the following oligonucleotide primer pairs: 1-A, 2-B, and 3-C (Fig. 1B). The PCR product confirmed CBS732 genome sequencing data. Interestingly, a PCR product was detected by using 1-B, 2-A and 3-C primer pairs, but not 1-A and 2-B primer pairs, in NBRC1130. This amplification pattern was maintained in ATCC2623. It should be noted that CBS732, NBRC1130 and ATCC2623 are the same strains grown using different culture collections. In addition, a PCR product was detected by using the primer pairs 1-A, 2-A, and 3-C in NBRC1733, and a PCR product was detected by using the primer pairs 1-C, 2-A, and 3-C in NBRC0686, NBRC0740 and NBRC1053 (Fig. 1B). These results suggest that the flanking sequences of *MAT*, *HML*, and *HMR* are highly variable in the *Z. rouxii* population. To interpret this result, we evaluated all possible combinations of the chromosome pieces to yield the PCR amplicons detected in Fig. 1B. As shown in Fig. 1C, the PCR results can be represented by six possible combinations, implying that chromosomal rearrangements occurred in the *Z. rouxii* population.

With some combination of primers, we could not detect amplicons, e.g., 2-B in NBRC0686. If the sequences of the target flanking regions are not conserved due to SNPs and/or polymorphisms among these strains, the primers could not anneal to the target sequences and would give rise to false negative results. To reduce this possibility, we performed the same PCR analysis by using 1′, 2′, 3′, A′, B′, and C′ primers, which were designed to the out side of the 1-A, 2-B, and 3-C primer pairs. The results were identical to those obtained above (data not shown).

To identify the idiomorph encoded at each *MAT*-like locus, PCR analysis was carried out using an **a** or α idiomorph-specific primer together with the corresponding A′, B′, or C′ primer that flanked the each *MAT*-like locus (Fig. S3A, Table S1). Using the A′ primer, **a** idiomorph-specific signals were detected in DL2, NBRC1130 (slightly), and ATCC2623, and α idiomorph-specific signals were detected in CBS732, DA2, NBRC1130, NBRC1733, NBRC0686, NBRC0740, and NBRC1053. Using the B′ primer, **a** idiomorph-specific signals were detected in CBS732, DA2, DL2, NBRC1130, and ATCC2623. Using the C′ primer, α idiomorph-specific signals were detected in all of the strains used in this study (Fig. S3B). These results suggest that each *MAT*-like locus has the corresponding idiomorph. The inferred idiomorph was noted in Fig. 1B. This discrimination between **a** and α idiomorphs was also supported by sequence data (see below).

### Translocation and reciprocal translocation among *MAT*, *HML*, and *HMR* loci

**Figure 2:**
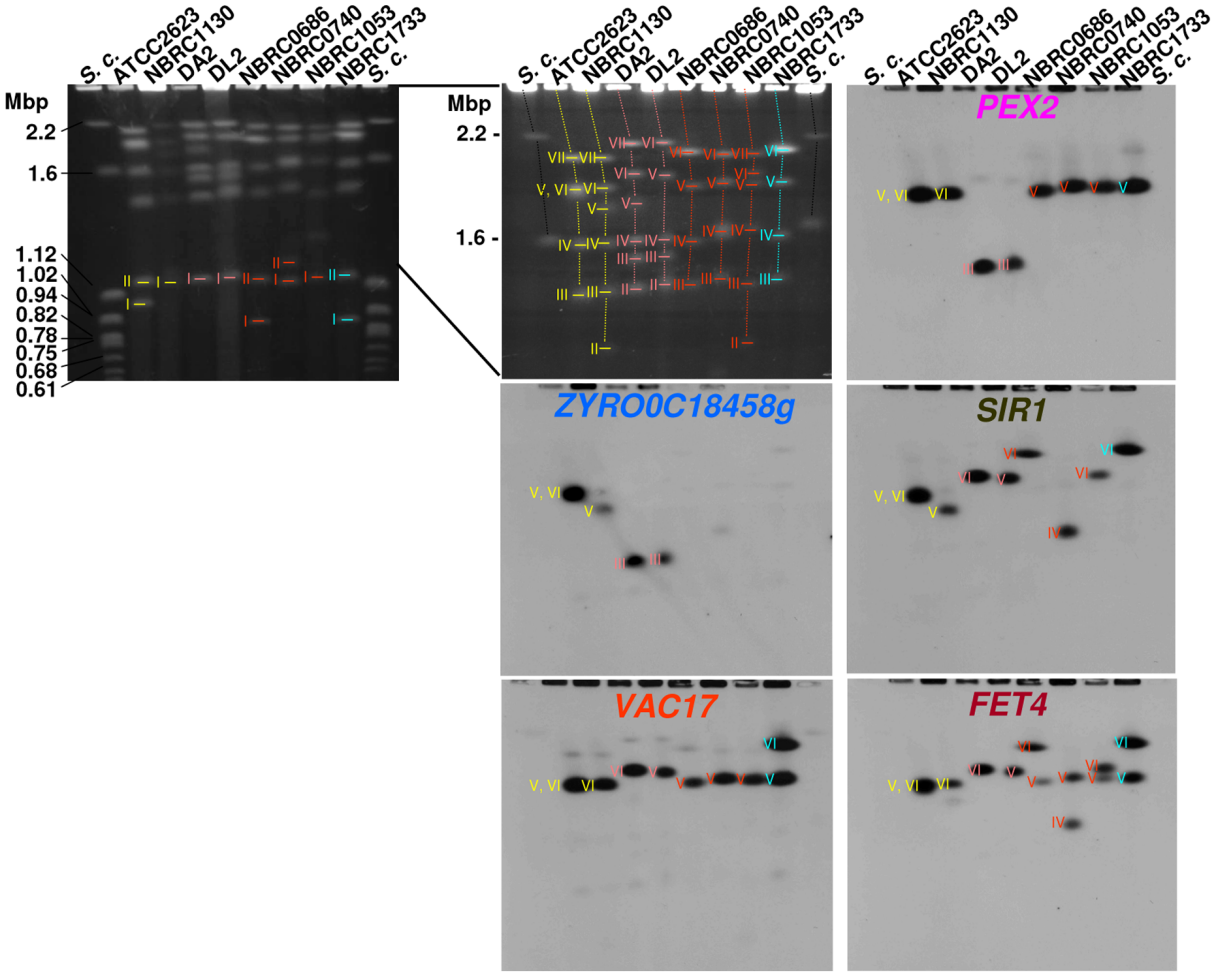
Mapping of *PEX2*, *ZYRO0C18458g*, *SIR1*, *VAC17*, and *FET4* by PFGE and Southern blot analysis. Chromosomal DNA was isolated from the following strains: ATCC2623, NBRC1130, DA2, DL2, NBRC0686, NBRC0740, NBRC1053, and NBRC1733. For Southern blot analysis, each ORF probe was used to map target genes. *S. c.* indicates *Saccharomyces cerevisiae* chromosome DNA marker. Chromosomal numbers are indicated by roman numerals.

To verify whether the high variability in the *MAT*, *HML*, and *HMR* flanking sequences in the studied yeast strains was accompanied by chromosomal rearrangement between the *MAT*, *HML*, and *HMR* loci, we compared the karyotype of these strains by using pulsed-field gel electrophoresis (PFGE). Although NBRC1130, DA2, DL2 and ATCC2623 share the same genetic background, their karyotypes were highly variable, suggesting that the karyotype is unstable in *Z. rouxii* (Fig. 2). Based on Southern blot analysis and genomic data from CBS732, *PEX2* and *ZYRO0C18458g* were located on chromosome 3, and *SIR1*, *VAC17*, and *FET4* were located on chromosome 6 and chromosome 5 in DA2 and DL2, respectively (Fig. 2). In NBRC1130, *PEX2*, *VAC17* and *FET4* were detected on chromosome 6, and *ZYRO0C18458g* and *SIR1* were detected together on chromosome 5. In NBRC0686, NBRC0740, and NBRC1053, two copies of *FET4*, one with *PEX2* and *VAC17* (on chromosome 5) and another with *SIR1* (on chromosomes 6, 4, and 6, respectively), were detected. *ZYRO0C18458g* was not detected in these strains. In NBRC1733, two copies of *VAC17* and *FET4*, one with *PEX2* (on chromosome 5) and another with *SIR1* (on chromosome 6), were detected, but *ZYRO0C18458g* was not detected (Fig. 2). These results suggest that the high variability within the *MAT*-flanking region is coincident with chromosomal rearrangements, which occurred by translocation or reciprocal translocation among the *MAT*-like loci. To determine the interchromosomal fusion point, we sequenced the PCR fragment from DA2, NBRC1130, NBRC1733, and NBRC0686 obtained by using specific primer sets designed outside of the 1-A, 2-B, and 3-C amplicons. Homologous recombination apparently occurred at the X or Z regions in the *MAT*, *HML*, and *HMR* loci; however, the precise fusion point could not be determined because these loci share nearly identical sequences in the X and Z regions. The sequences reported in this paper have been deposited in the DNA Data Bank of Japan (accession no. AB781017-AB781029), and some representative sequence data were shown in Fig. S5, S6, S7, S8, S9, and S10.

In ATCC2623, all signals from *PEX2*, *ZYRO0C18458g*, *SIR1*, *VAC17*, and *FET4* were unexpectedly detected at the same position (Fig. 2). These results suggest that these genes are located on the same chromosome or on different chromosomes that are similar in length. The PCR amplification pattern in ATCC2623 verified that the *MAT* organization in the ATCC2623 strain corresponded to the *MAT* organization found in NBRC1130 (Fig. 1B).

### The original organization of the *MAT* locus in *Z. rouxii*

**Figure 3:**
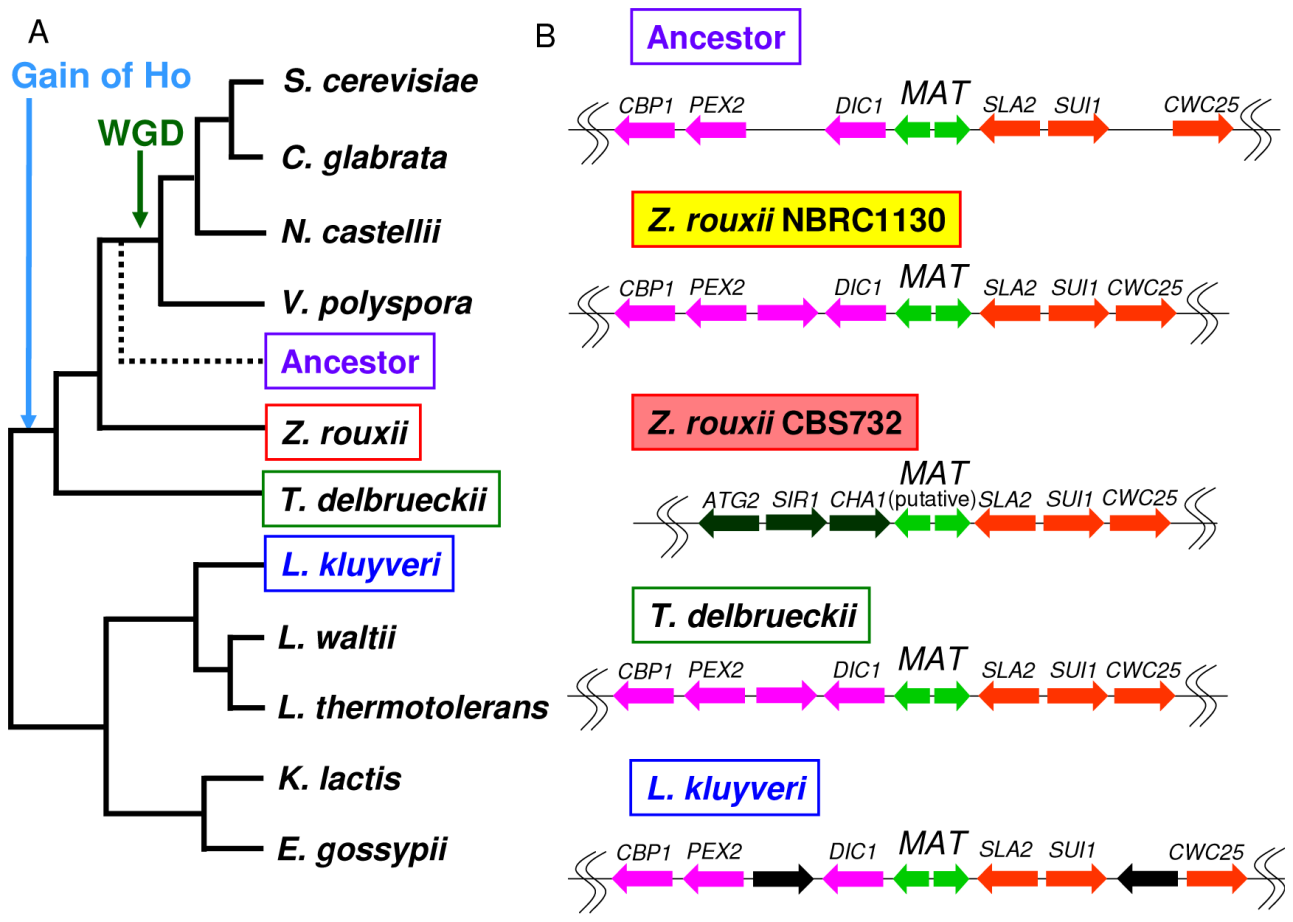
The original organization around the *MAT* locus in *Z. rouxii*. (A) Phylogenic relationships among the species and the position of the reconstructed ancestral genome. The phylogenic tree is not to scale, and the topology is derived from a referenced study [29] (B) The gene organization around the *MAT* locus from representative yeasts before and after *HO* gene acquisition.

**Figure 4:**
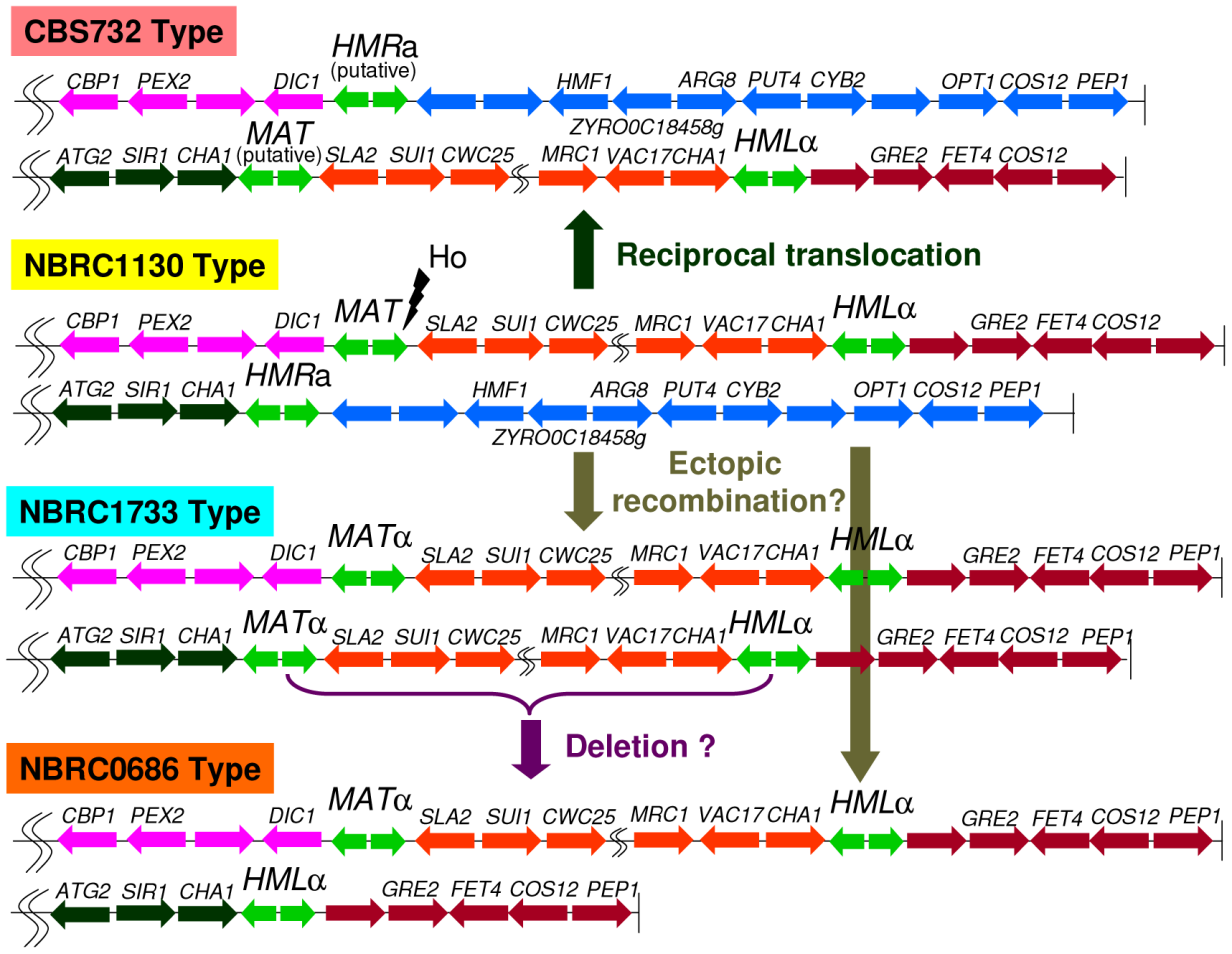
Inferred comparative organization around the *MAT*-like locus in *Z. rouxii*. Coloring indicates gene position in NBRC1130: pink, left side of *MAT*; orange, between *MAT* and *HML*; wine red, right side of *HML*; dark green, left side of *HMR*; and blue, right side of *HMR*. The vertical bars are telomeres. The gene order was predicted from CBS732 except for around the fusion points.

One of the significant findings from this study is the high variability in the *MAT* locus detected in NBRC1130, DL2, DA2 and ATCC2623, despite the fact that these strains originated from the same strain. To determine the original organization of the *MAT* locus in *Z. rouxii*, we compared the *MAT* organization with the gene organization in related species. Gordon *et al*. inferred the gene order and core genomic structure of the ancestral species that existed immediately before WGD [16]. This ancestral strain and *Lachancea kluyveri*, which diverged from the *Saccharomyces* lineage before the divergence of *Z. rouxii*, have the same gene order around the *MAT* locus in NBRC1130; *DIC1* is located to the left of *MAT*, and *SLA2* is located to the right of *MAT* in both species. A similar organization (*DIC1*-*MAT*-*SLA2*) is also observed in *Torulaspora delbrueckii*, which has a closely related genome to *Z. rouxii* [4]. CBS732 and its derivative also have *SLA2* to the right of *MAT*, whereas *CHA1* is located on the left side of *MAT* (Fig. 3A, B). These results suggest that the organization of the *MAT* locus in NBRC1130 is original, unlike the organization of CBS732, which was used for genomic sequencing. The chromosomal organization of CBS732 could have been caused by reciprocal translocation between *MAT* and *HMR* (Fig. 4). We designated the *MAT*-like locus (dark green-orange junction) in CBS732 as the putative *MAT* locus because mating type switching was observed at the locus (Fig. 1, Fig. S3).

The organization of NBRC0686, NBRC0740, NBRC1053, and NBRC1733 could have been caused by interchromosomal rearrangement accompanying ectopic recombination between the *MAT* and *HMR* loci or between the *HML* and *HMR* loci (Fig. 4). Accordingly, the region containing *ZYRO0C18458g* to the right of the *HMR* locus is missing in NBRC0686, NBRC0740, NBRC1053, and NBRC1733.

### Translocation frequency

**Figure 5:**
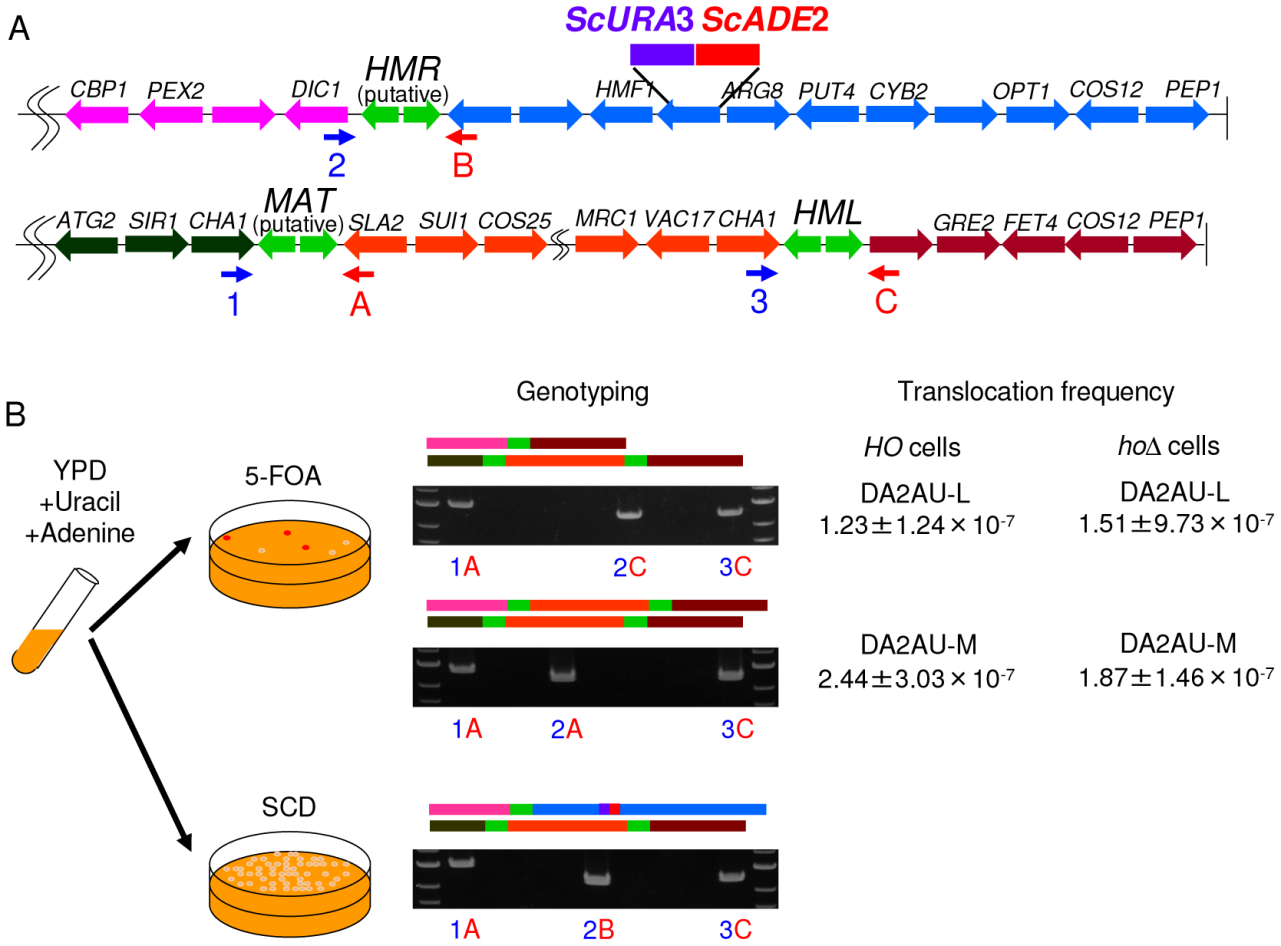
The frequency of translocation. (A) Gene organization around the *MAT*-like loci in *Z. rouxii* strain DA2AU. The *ScURA3* and *ScADE2* genes were inserted into the *ZYRO0C18458g* locus in DA2. The vertical bars are telomeres. (B) Assay system to screen for spontaneous mutants that lead to ectopic recombination. DA2AU was incubated in YPD supplemented with adenine and uracil for 42 h at 30°C and then transferred to 5-FOA and YPD plates. The medium was incubated for 2 weeks at 30°C. The genotypes of red colonies recovered on 5-FOA plates were checked by PCR, and the translocation frequency was calculated by the following equation: (number of strains with a translocation)/(colony formation units on YPD plate).

Why does *Z. rouxii* have a highly variable *MAT* organization? To determine the translocation frequency between *MAT* and *HMR* or between *HML* and *HMR*, we constructed the DA2AU strain, which has *ScADE2* and *ScURA3* at the *ZYRO0C18458g* locus in the DA2 and DA2 *ho*Δ backgrounds. If the right side of *HMR* is lost and replaced by the right side of *MAT* or *HML* (designated DA2AU-M or DA2AU-L mutants, respectively), we can positively select the mutants as red colonies on 5-FOA plates (Fig. 5).

We obtained a total of 175 5-FOA^r^ red colonies from 3.96×10^8^ screenings in *HO* cells and 169 5-FOA^r^ red colonies from 8.17×10^8^ screenings in *ho*Δ cells. The resulting colonies were classified by PCR using the primer set used in Fig. 1. In *HO* cells, a total of 106 DA2AU-M and 61 DA2AU-L mutant colonies were identified, and the remaining 4 colonies could not be classified. In *ho*Δ cells, a total of 92 DA2AU-M and 76 DA2AU-L mutant colonies were identified, and the remaining 1 colony could not be classified. In *HO* cells, the frequencies of spontaneous mutation were 2.44±3.03×10^−7^ and 1.23±1.24×10^−7^ for DA2AU-M and DA2AU-L, respectively (Fig. 5). In *ho*Δ cells, the frequencies of spontaneous mutation were 1.51±9.73×10^−7^ and 1.87±1.46×10^−7^ for DA2AU-M and DA2AU-L, respectively (Fig. 5). No significant differences were observed between the DA2 and DA2 *ho*Δ backgrounds. To verify that the *HO* gene catalyzed DSBs and enabled efficient switching, semi-quantitative PCR was carried out using an **a** or α idiomorph-specific primer together with a primer from the common flanking sequence (Table S1). The flanking primer resided outside the W and Z regions shared with the *HMR* and *HML* cassettes and, therefore, will only amplify a product from the *MAT* locus. The α idiomorph-specific PCR products were detected at cycles 27 and 24 in DA2 and DA2 *ho*Δ, respectively, whereas the **a** idiomorph-specific PCR products were detected at cycles 33 and 36 in DA2 and DA2 *ho*Δ, respectively. From these results, the mating type conversion ratio in DA2 was at least 8-fold higher than the conversion ratio in DA2 *ho*Δ (Fig. S4). These results indicate that the acquisition of HO endonuclease increased the frequency of genotypic switching in *Z. rouxii*; however, the interchromosomal rearrangement accompanying ectopic recombination between *MAT* and *HMR* or between *HML* and *HMR* is independent of HO endonuclease at least under our experimental conditions.

## Discussion

We suggest that the original organization of the *MAT* locus is represented in NBRC1130, and the organization of the *MAT* locus in CBS732 was made by reciprocal translocation between *MAT* and *HMR* during the recovery from occasional accidents that occurred during mating-type switching. The reciprocal translocation events may resemble the fusion of *MAT* with *HMR* **a** (Hawthorne's deletion) and of *MAT* with *HML*α (alpha-ring chromosome) in *S. cerevisiae* [17], [18]. Perhaps this reciprocal translocation occurred in the early passage culture of CBS732 during the past 80 years because the type strain, deposited in the Centraalbureau voor Schimmelcultures (CBS) collection and distributed to other collections, was originally isolated by Sacchetti in 1932 [19]; the *MAT* locus organization of these other collections (ATCC2623 and NBRC1130) is now different from that of CBS732. Unfortunately, the CBS732 strain was used for genomic sequencing of *Z. rouxii* [8] even though the *MAT* locus organization of CBS732 is not the original. Gordon *et al*. found that translocation had joined the X side of the *MAT* locus (left side of *MAT* in Fig. 1A) to a telomeric region containing *CHA1* in *Z. rouxii* [4]; however, this reciprocal translocation is a CBS732-specific event and is not common in the *Z. rouxii* population.

PCR products amplified using oligonucleotide primer pair 1-A were α and **a** in DA2 and DL2, respectively (Fig. 1B, S2B). In addition to the α-specific PCR product, an **a**-specific PCR product was also detected in DA2 (Fig. S4), indicating that mating type conversion occurred at the “new” *MAT*-like locus (dark green-orange junction) in CBS732 and its derivatives. This fact leads us to infer that the new *MAT*-like locus is the *MAT* locus. In this scenario, one of the products (dark green-orange junction) must become active, and the other one (pink-blue junction) must become silenced. How do the loci change between an active and a silenced state? We hypothesize 3 possible reasons: 1) the distance from the telomere determines the silencing state, i. e., the locus labeled *MAT* (putative) is active because its location is far from the telomere, and the locus labeled *HMR* (putative) is silenced because its location is within a subtelomerers where genes are transcriptionally silenced, 2) cis-acting silencers, known as *E* and *I* in *S. cerevisiae*, lie in to the right side of *HMR* (blue region), or 3) both situations are true. To obtain solid evidence to demonstrate that the *MAT*-like locus (dark green-orange junction) is *MAT*, we tried to obtain a zygote between DA2 (*ura3* ^−^ *ade2* ^−^) and DL2 (*ura3* ^−^ *leu2* ^−^) many times; however, we could not obtain a zygote with only uracil auxotrophy. Taken together, we have no direct evidence to conclude that the locus labeled *MAT* is actually active *MAT*, and thus, a study that is more detailed is needed.

PCR products from 2-A in NBRC1130 were **a**/α, indicating either diploids or a mixture of *MAT* **a** and *MAT*α. However, the **a**/α ratio estimated from PCR signal intensity was not reproducible in NBRC1130; PCR analysis detected only **a** amplicons some of the time, only α amplicons some of the time, or an **a**/α mixture (or diploid) some of the time. Perhaps, the **a**/α ratio in the initial single colony, which was precultured for genomic DNA extraction, caused the poor reproducibility. We tried to identify a stable diploid from the colony which gave a mixture of equal parts of **a** and α using PCR analysis; however, we failed to recover a stable diploid. In any case, CBS732 and NBRC1130 were potentially homothallic, in accordance with the classical observation that sugar-tolerant strains have a mating response in each culture without mixing with any other strains [20]. Interestingly, NBRC0686, NBRC0740, NBRC1053, and NBRC1733 lost **a** information, indicating that these strains were obligate heterothallic. Natural **a**,**a**,**a** and α,α,α isolates of *S. cerevisiae* are found at a low frequency [21], and an α,α,α isolate of *Candida grabrata* has been described [22]. In *Z. rouxii*, an α,α,α isolate (NBRC0686, NBRC0740 and NBRC1053) and an α,α,α,α isolate (NBRC1733) could be caused by the interchromosomal rearrangement accompanying ectopic recombination between *MAT* and *HMR* or between *HML* and *HMR* and/or subsequent mating-type switching.

Under our experimental condition, the frequency of switching in *HO* cells was at least 8 times higher than in *ho*Δ cells in *Z. rouxii* (Fig. S4). These results indicate that the acquisition of HO endonuclease increased the frequency of genotypic switching in *Z. rouxii*. However, the frequency of switching is 10^6^-times higher in *HO* than in *ho*Δ cells in *S. cerevisiae* [23], indicating that the contribution of HO endonuclease to genotypic switching varies among yeast species. The genotype switching from *MAT*α to *MAT*a still occurred at low frequency in *ho*Δ cells. In the genus *Kluyveromyces*, it is known that the genotype switching from *MAT*α to *MAT*a is induced by α3 gene excisition-dependent DSBs and the opposite direction switching from *MAT*a to *MAT*α is occurred by a different but uncharacterized mechanism [24], [25]. It is therefore that the switching observed in *ho*Δ cells in this study may be occurred by an uncharacterized mechanism similar to genus *Kluyveromyces*.

We analyzed spontaneous translocation events in DA2AU strains that led to functional inactivation of a *URA3* and *ADE2* marker inserted to the right of the *HMR* locus. The frequencies of spontaneous translocation were approximately 10^−7^, and no significant differences were observed between *HO* cells and *ho*Δ cells. The HO endonuclease recognition site of *HMR* is blocked by chromatin modification in many hemiascomycetous yeast [26]. Therefore, the translocations observed in NBRC0686, NBRC0740, NBRC1053 and NBRC1733 are caused by interchromosomal rearrangement using homologous recombination and crossing over at the X and Z regions in the *MAT*-like locus.

In this study, we analyzed diversity at the mating-type locus in the *Z. rouxii* population and demonstrated that the *MAT* and *MAT*-like loci were susceptible regions in the *Z. rouxii* genome. To explore the precise evolution of the *MAT* locus, investigation of the intraspecies mutations at the *MAT* locus in various yeast species is needed.

## Materials and Methods

### Strains, media, and genetic methods

The yeast strains used in this study are listed in Table 1. *Z. rouxii* cells were transformed using an improved electroporation method [27]. Standard YPD medium containing G418 and synthetic complete dextrose (SCD) medium lacking adenine and/or uracil for transformant selection were used.

**Table 1.Yeast:** strains used in this study.

Species	Strain^a^	Other collection numbers	*Source of isolate/reference*
*Z. rouxii*	CBS732^T^	ATCC2623, NBRC1130	*Black grape must concentrate*
	DA2		*Derived from CBS732 * [*14*] b
	DL2		*Derived from CBS732 * [*14*] b
	NBRC1130^T^	ATCC2623, CBS732	*Black grape must concentrate*
	ATCC2623^T^	CBS732, NBRC1130	*Black grape must concentrate*
	NBRC0686	CBS741, NCYC581	*Honey, Canada*
	NBRC0740	CBS2752	*Sputum, Norway*
	NBRC1053	CBS7412, NCYC170	*Sugar solution containing ginger rhizome*
	NBRC1733	ATCC8381, CBS742	*Fermenting honey, Canada*
	NBRC1876	CBS4837, NCYC1682, NRRL Y-2547	*Miso paste, Japan*
	NBRC1877	CBS4838, NRRL Y-2548	*Miso paste, Japan*
	Z3		*Soy sauce * [*27*]
	DA2AU		*Derived from DA2 [This study]*
	DA2AU*ho*Δ		*Derived from DA2AU [This study]*
	DA2*ho*Δ		*Derived from DA2 [This study]*
*S. cerevisiae*	BY4742		
	*BY4743*		

a T, type strain.

b Gift from Hana Sychrová.

### PCR and sequence analysis

*MAT*-like loci were amplified from the chromosomal DNA by using primers 1, 2, 3, A, B, and C, or 1′, 2′, 3′, A′, B′, and C′ (Table S1). Each PCR reaction mixture (50 µl) consisted of 1 µl of DNA (approximately 10 ng), 25 µl of 2X KOD-FX neo buffer (Toyobo, Osaka, Japan), 0.4 mM of each deoxynucleoside triphosphate (Toyobo), 25 pmol of each primer, and 1 unit of KOD-FX neo DNA polymerase (Toyobo). PCR was performed for 33 cycles after a pre-incubation at 94°C for 2 min using the following conditions: denaturation at 98°C for 10 s; annealing at 57°C for 15 s; extension at 68°C for 2 min. The amplicons were analyzed by agarose gel electrophoresis.

To obtain sequence information of the *MAT*-like loci, the PCR products were purified using the Wizard SV Gel and PCR Clean-Up system (Promega, Fitchburg, USA) according to the supplier's instructions. Single-extension sequencing from both strands of DNA products was performed by a commercial DNA sequencing service provider (Greiner-Japan, Tokyo, Japan).

To identify the genotype (**a** or α), a fragment containing partial **a** or α idiomorphs was amplified by PCR from the chromosomal DNA using the primers MTLaF or MTLαF, and A′, B′, or C′. PCR was performed for 33 cycles after a pre-incubation at 94°C for 2 min using the following conditions: denaturation at 98°C for 10 s; annealing at 58°C for 15 s; extension at 68°C for 30 s.

### Flow cytometry

Flow cytometry was carried out as described by Solieri *et al*. [11] with little modification. Briefly, the strains were grown to the mid-log phase (OD_600 mm_ of 0.5 to 0.6) in SCD (5% glucose) medium at 28°C with shaking. Cell samples were induced to arrest at the G1 phase by treatment with 8-hydroxyquinoline at a final concentration of 100 mg/l for 24 h. Cells were harvested by centrifugation (3,000×*g* for 3 min at 4°C) and fixed overnight with 70% (v/v) ethanol at 4°C. After fixation, cells were harvested, washed, and resuspended at a concentration of 5×10^6^ cells/ml in 50 mM sodium citrate buffer, pH 7.0. The cell suspension was treated for 120 min at 50°C with RNase A and proteinase K at final concentrations of 0.25 and 2 mg/ml, respectively. A 2.5×10^6^ cells/ml cell suspension was stained overnight at 4°C with propidium iodide at a final concentration of 4 µg/ml. To remove clumps and debris, sonic pulses were applied. Before FCM analysis, cells were filtered through a 35 µm mesh cell-strainer (Becton, Dickinson and Company, Franklin Lakes, NJ). FCM experiments were performed on an Accuri™ C6 flow cytometer (Becton, Dickinson and Company). A minimum of 20,000 cells per sample was acquired at a low flow rate. Offline data were analyzed with the CFlow plus software (Becton, Dickinson and Company).

### Chromosomal DNA isolation and PFGE

Chromosomal DNA in a plug was prepared according to the protocol used in a previous study with minor modifications [28]. Zymolyase was used instead of lyticase, and cell treatment was performed overnight at 30°C. One percent agarose gels were prepared using pulsed-field certified agarose (Bio-Rad, Hercules, CA) and run on a CHEF apparatus (Bio-Rad) in 0.5× TBE buffer at 10°C. The following running conditions were used: switch time, 300–400 s or 400 s; run time, 120 h or 180 h; angle, 120°; and voltage, 3 V/cm.

### Southern blot analysis

Chromosomal DNAs separated by PFGE were transferred onto a Hybond-N^+^ membrane (GE Healthcare, Buckinghamshire, UK) by upward capillary transfer. Open-reading frame regions of *PEX2*, *ZYRO0C18458g*, *SIR1*, *VAC17*, and *FET4* genes were then used to probe the membranes. These experiments were performed using a PCR digoxigenin probe synthesis kit (Roche, Basel, Switzerland) and Detection Starter Kit II (Roche) according to the manufacturer's instructions.

### Estimation of switching frequency

The frequency of switching was estimated by the semi-quantitative PCR. A fragment containing partial **a** or α idiomorphs was amplified by PCR from the chromosomal DNA of DA2 and DA2 *ho*Δ cells using the primers MTLaF or MTLαF and TypingR. PCR was performed for 39 cycles after a pre-incubation at 94°C for 2 min using the following conditions: denaturation at 98°C for 10 s; annealing at 58°C for 15 s; extension at 68°C for 30 s.

### Estimation of spontaneous translocation frequency

The DA2AU strain was constructed by inserting *S. cerevisiae URA3* and *ADE2* into the *ZYRO0C18458g* locus of DA2. The *ZYRO0C18458g* locus was amplified from DA2 using the primers C18458gF and C18458gR, and the resulting fragment was cloned into pT7blue (Takara Bio, Siga, Japan), which was designated pTC1. pZEU [14] was digested with *Bgl*II, and the resulting fragment containing *URA3* was inserted into the *Bgl*II restriction site of pTC1. To introduce *URA3* into the *ZYRO0C18458g* locus, we amplified this plasmid using primers C18458gF and C18458gR, and the resulting fragment was used for transformation. To introduce *ADE2* into the *ZYRO0C18458g* locus, we amplified *ADE2* from *S. cerevisiae* BY4743 using primers ScADE2InsF and ScADE2InsR, and the resulting fragment was used for transformation. The resulting transformant was designated DA2AU. To construct *ho* cells, we amplified G418-resistant genes from pCUG using the primers HOdisF and HOdisR, and the resulting fragment was used for transformation of DA2. To quantify the frequency of translocation, the DA2AU strain was incubated with agitation in YPD supplemented with adenine and uracil for 42 h at 30°C, then transferred to a 5-FOA plate (SCD plate supplemented with 0.2% 5-fluoroorotic acid), and incubated for 2 weeks at 30°C. Red colonies, which can grow on this 5-FOA plate, were selected and transferred to fresh same medium, and the resulting red colonies were genotyped by PCR analysis. The relative difference between the translocated strain and colony formation number on the YPD plate was used to determine the translocation frequency.

## Supporting Information

Figure S1 **Isolation of a CBS732-like haploid strain in** ***Z. rouxii*** **.** (A) Summary of natural hybridization events between haploid *Z. rouxii* and *Z. pseudorouxii*. The orange and green bars indicate the T and P subgenomes, respectively. (B) Selective amplification of the *ADE2* and *SOD2* genes. The *SOD2* gene encoded in the T and P subgenomes has been referred to as *Zr-SOD2-22* and *Zr-SOD22*, respectively [30], [31].
(TIF)

Figure S2 **Fluorescence histograms of various** ***Z. rouxii*** **strains after propidium iodide staining.** The y-axis in the graphs represents the total cell counts, and the x-axis indicates the relative fluorescence intensity of the samples.
(TIF)

Figure S3 **Idiomorph carring at the** ***MAT*** **-like loci.** (A) Gene organization around the *MAT*-like locus. Small arrows indicate primers. (B) PCR amplification of *MAT*-like loci from *Z. rouxii* CBS732, DA2, DL2, NBRC1130, ATCC2623, NBRC1733, NBRC0686, NBRC0740, and NBRC1053. PCR was performed by using idiomorph-specific primers and primers specific to each locus (Table S1).
(TIF)

Figure S4 **Semi-quantitative PCR amplification of idiomorph-specific products from** ***Z. rouxii*** **DA2 and DA2 h** ***o*** **Δ.** PCR was performed by using idiomorph-specific primers, and genomic DNA from DA2 and DA2 *ho*Δwas used as a template.
(TIF)

Figure S5 **Nucleotide sequence of the putative** ***MAT*** **locus amplified by using primer pair 1′-A′ from DA2 (AB781017).** Uppercase lettering indicates putative open reading frame. Arrows indicate gene direction. Coloring indicates gene position in NBRC1130: dark green, left side of *HMR*; gray, X and Z region; and orange, between *MAT* and *HML*.
(TIF)

Figure S6 **Nucleotide sequence of the** ***HMR*** **locus amplified by using primer pair 1′-B′ from NBRC1130 (AB781022).** Uppercase lettering indicates putative open reading frame. Arrows indicate gene direction. Coloring indicates gene position in NBRC1130: dark green, left side of *HMR*; gray, X and Z region; and light blue, right side of *HMR*.
(TIF)

Figure S7 **Nucleotide sequence of the** ***HML*** **locus amplified by using primer pair 1′-C′ from NBRC0686 (AB781028).** Uppercase lettering indicates putative open reading frame. Arrows indicate gene direction. Coloring indicates gene position in NBRC1130: dark green, left side of *HMR*; gray, X and Z region; and wine red, right side of *HML*.
(TIF)

Figure S8 **Nucleotide sequence of the** ***MAT*** **locus amplified by using primer pair 2′-A′ from NBRC1130 (AB781020).** Uppercase lettering indicates putative open reading frame. Arrows indicate gene direction. Coloring indicates gene position in NBRC1130: pink, left side of *MAT*; gray, X and Z region; and orange, between *MAT* and *HML*.
(TIF)

Figure S9 **Nucleotide sequence of the putative** ***HMR*** **locus amplified by using primer pair 2′-B′ from DA2 (AB791018).** Uppercase lettering indicates putative open reading frame. Arrows indicate gene direction. Coloring indicates gene position in NBRC1130: pink, left side of *MAT*; gray, X and Z region; and light blue, right side of *HMR*.
(TIF)

Figure S10 **Nucleotide sequence of the putative** ***HML*** **locus amplified by using primer pair 3′-C′ from DA2 (AB781019).** Uppercase lettering indicates putative open reading frame. Arrows indicate gene direction. Coloring indicates gene position in NBRC1130: orange, between *MAT* and *HML*; gray, X and Z region; and wine red, right side of *HML*.
(TIF)

Table S1 **Primers used in this study.**
(XLS)
