# Supplementary figures and images for: Diversity of Mating-Type Chromosome Structures in the Yeast Zygosaccharomyces rouxii Caused by Ectopic Exchanges between MAT-Like Loci
Source: PLoS One. 2013 Apr 16;8(4):e62121. doi: 10.1371/journal.pone.0062121 (PMC3628578; doi:10.1371/journal.pone.0062121)

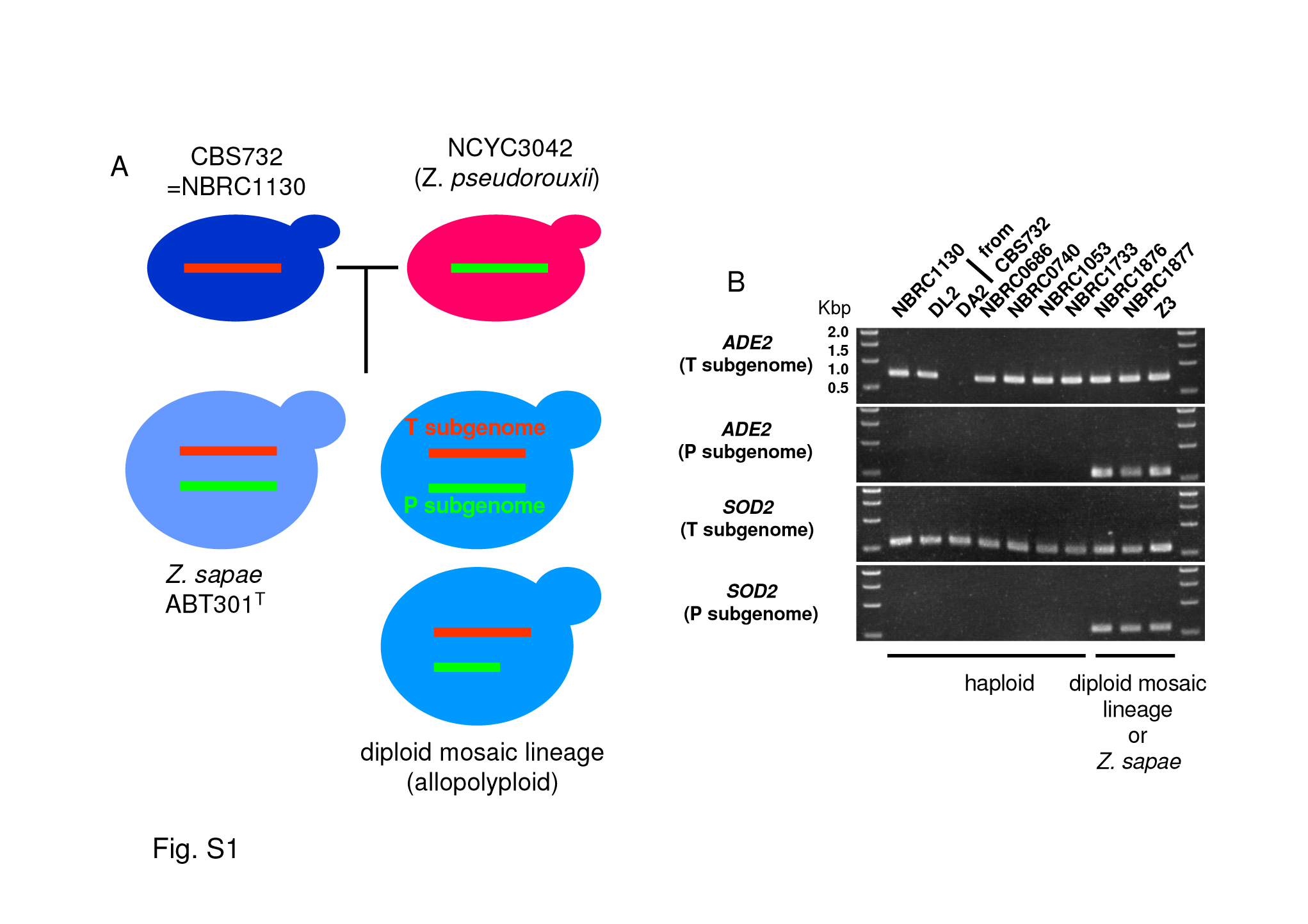

Supplement: Figure S1 — Isolation of a CBS732-like haploid strain in Z. rouxii . (A) Summary of natural hybridization events between haploid Z. rouxii and Z. pseudorouxii. The orange and green bars indicate the T and P subgenomes, respectively. (B) Selective amplification of the ADE2 and SOD2 genes. The SOD2 gene encoded in the T and P subgenomes has been referred to as Zr-SOD2-22 and Zr-SOD22, respectively [30], [31]. (TIF) [file pone.0062121.s001.tif]

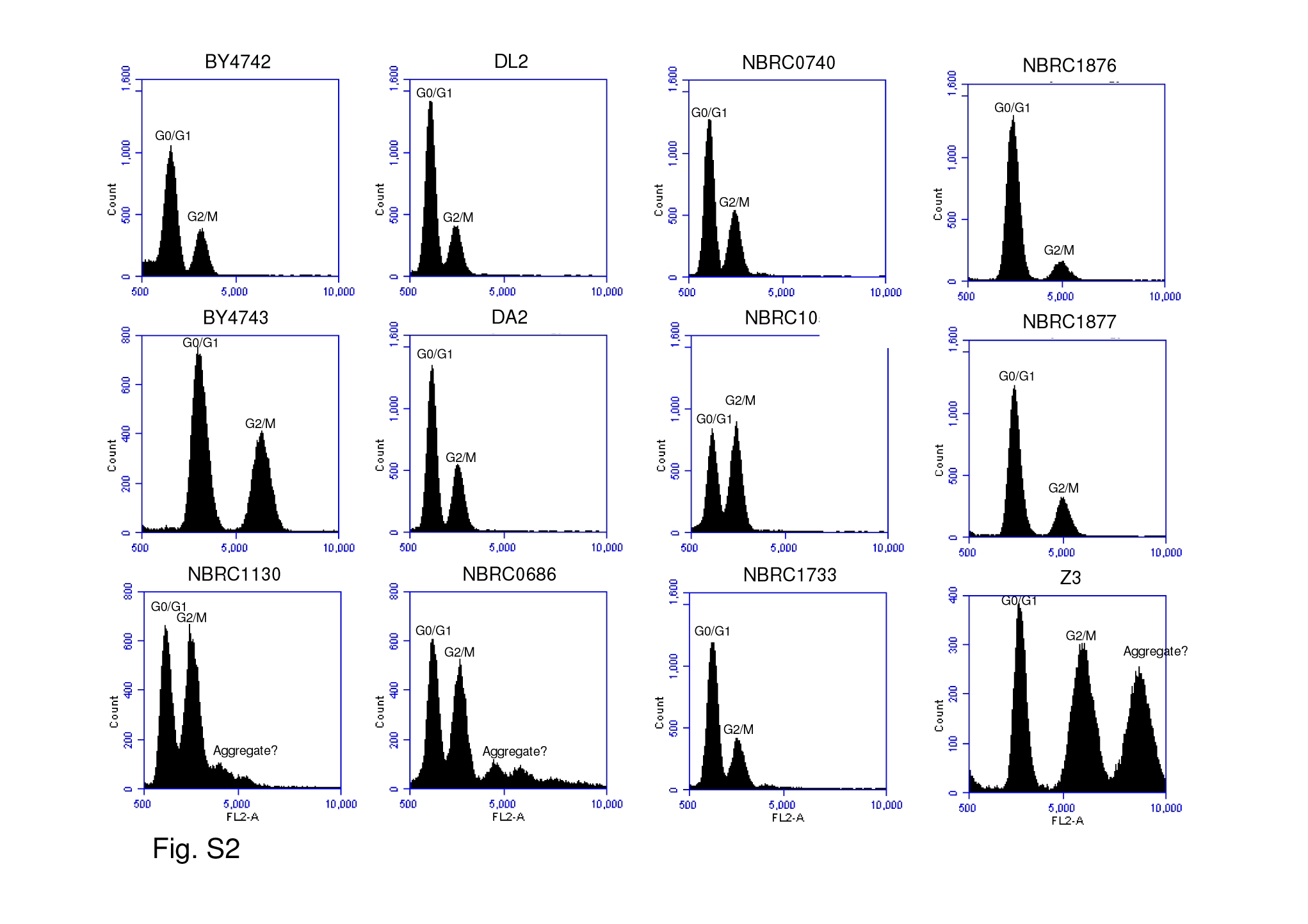

Supplement: Figure S2 — Fluorescence histograms of various Z. rouxii strains after propidium iodide staining. The y-axis in the graphs represents the total cell counts, and the x-axis indicates the relative fluorescence intensity of the samples. (TIF) [file pone.0062121.s002.tif]

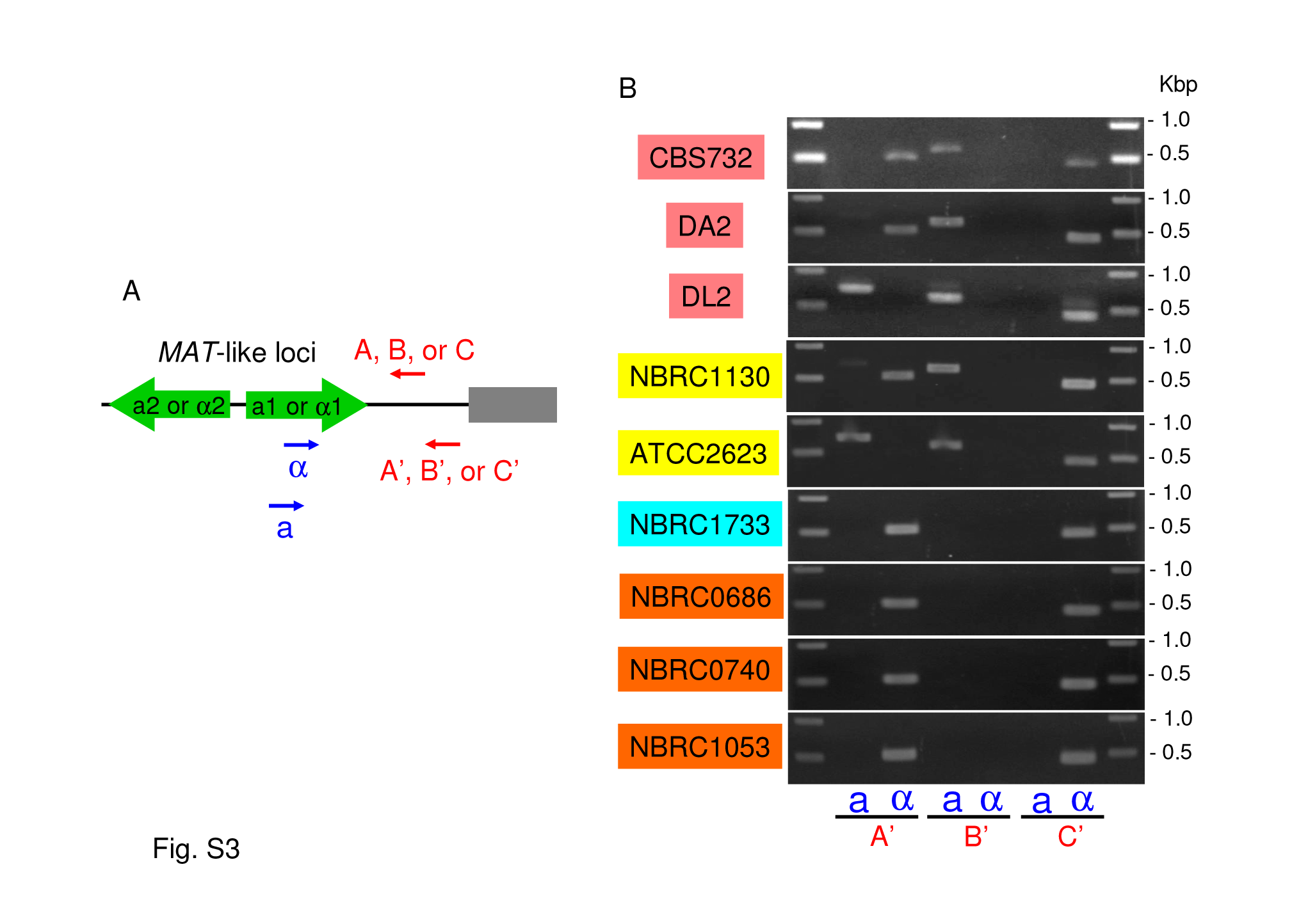

Supplement: Figure S3 — Idiomorph carring at the MAT -like loci. (A) Gene organization around the MAT-like locus. Small arrows indicate primers. (B) PCR amplification of MAT-like loci from Z. rouxii CBS732, DA2, DL2, NBRC1130, ATCC2623, NBRC1733, NBRC0686, NBRC0740, and NBRC1053. PCR was performed by using idiomorph-specific primers and primers specific to each locus (Table S1). (TIF) [file pone.0062121.s003.tif]

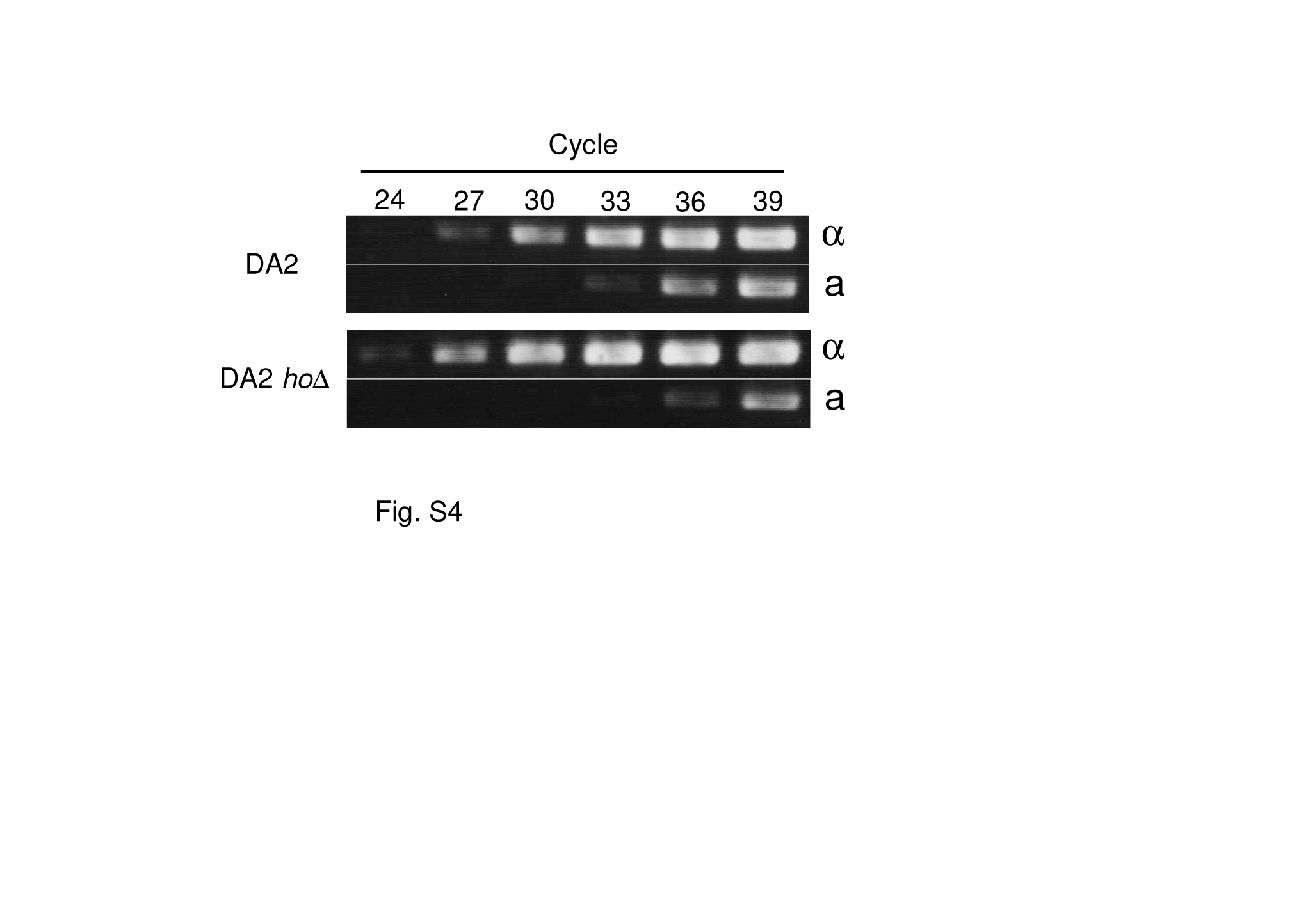

Supplement: Figure S4 — Semi-quantitative PCR amplification of idiomorph-specific products from Z. rouxii DA2 and DA2 h o Δ. PCR was performed by using idiomorph-specific primers, and genomic DNA from DA2 and DA2 hoΔwas used as a template. (TIF) [file pone.0062121.s004.tif]

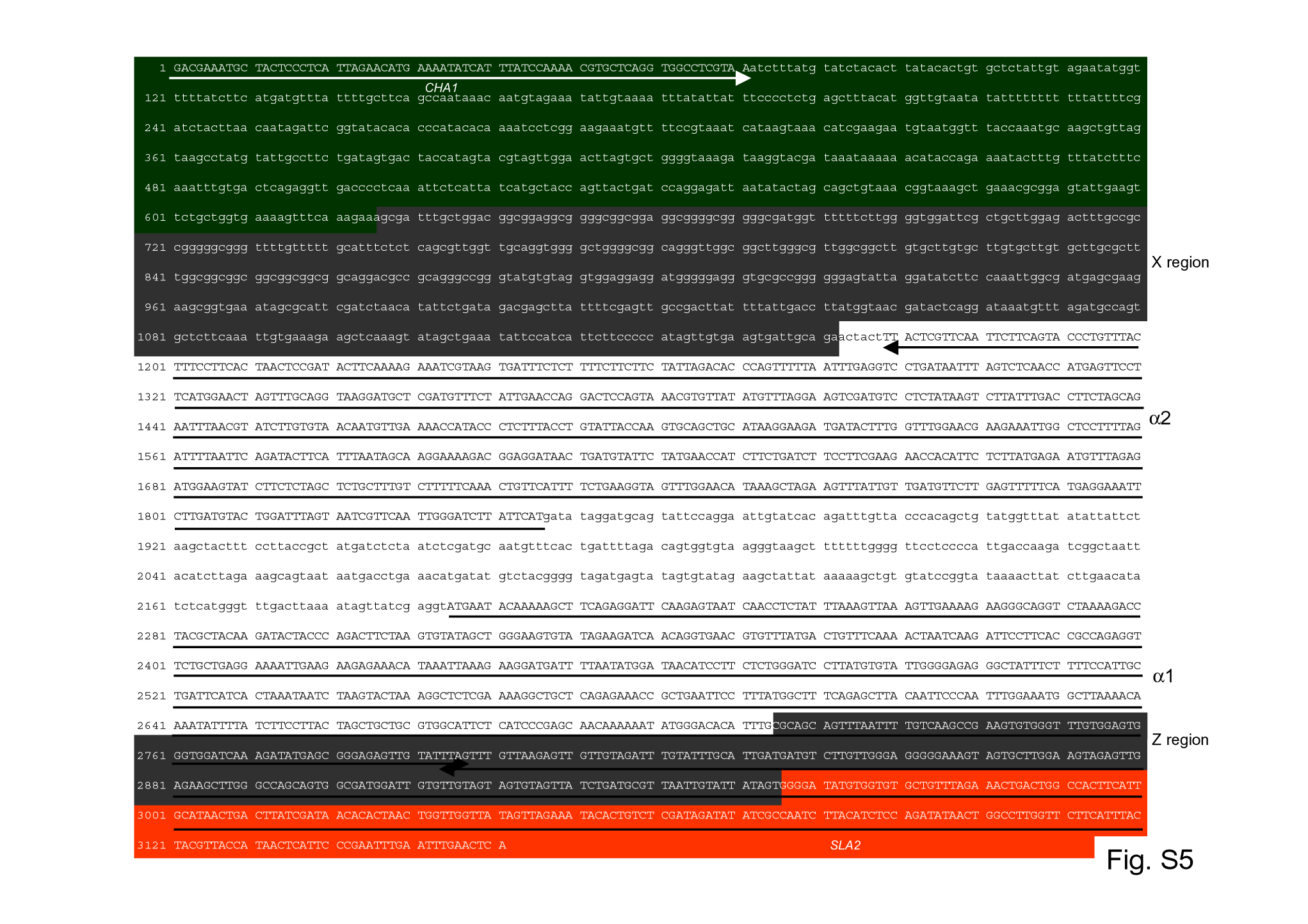

Supplement: Figure S5 — Nucleotide sequence of the putative MAT locus amplified by using primer pair 1′-A′ from DA2 (AB781017). Uppercase lettering indicates putative open reading frame. Arrows indicate gene direction. Coloring indicates gene position in NBRC1130: dark green, left side of HMR; gray, X and Z region; and orange, between MAT and HML. (TIF) [file pone.0062121.s005.tif]

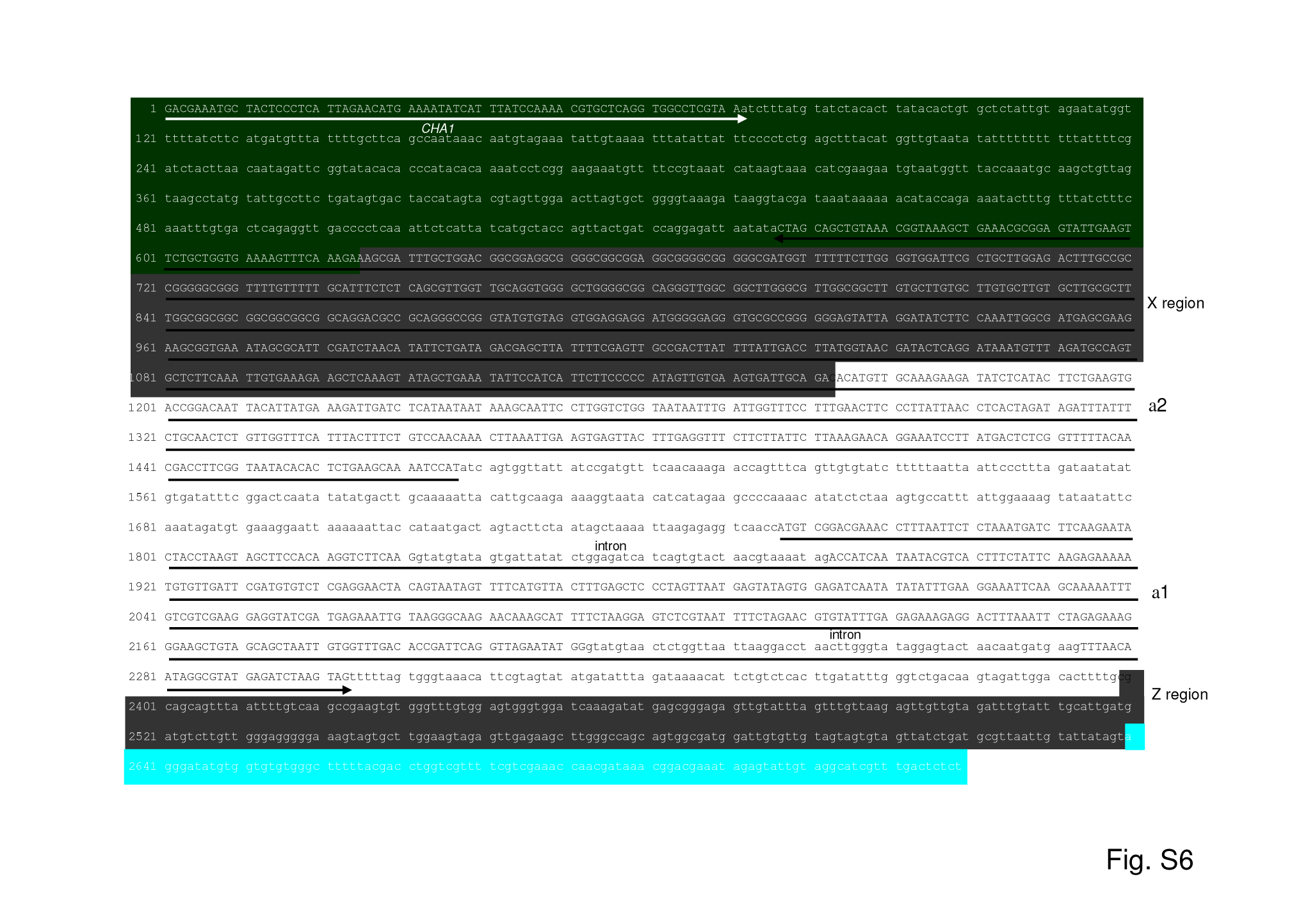

Supplement: Figure S6 — Nucleotide sequence of the HMR locus amplified by using primer pair 1′-B′ from NBRC1130 (AB781022). Uppercase lettering indicates putative open reading frame. Arrows indicate gene direction. Coloring indicates gene position in NBRC1130: dark green, left side of HMR; gray, X and Z region; and light blue, right side of HMR. (TIF) [file pone.0062121.s006.tif]

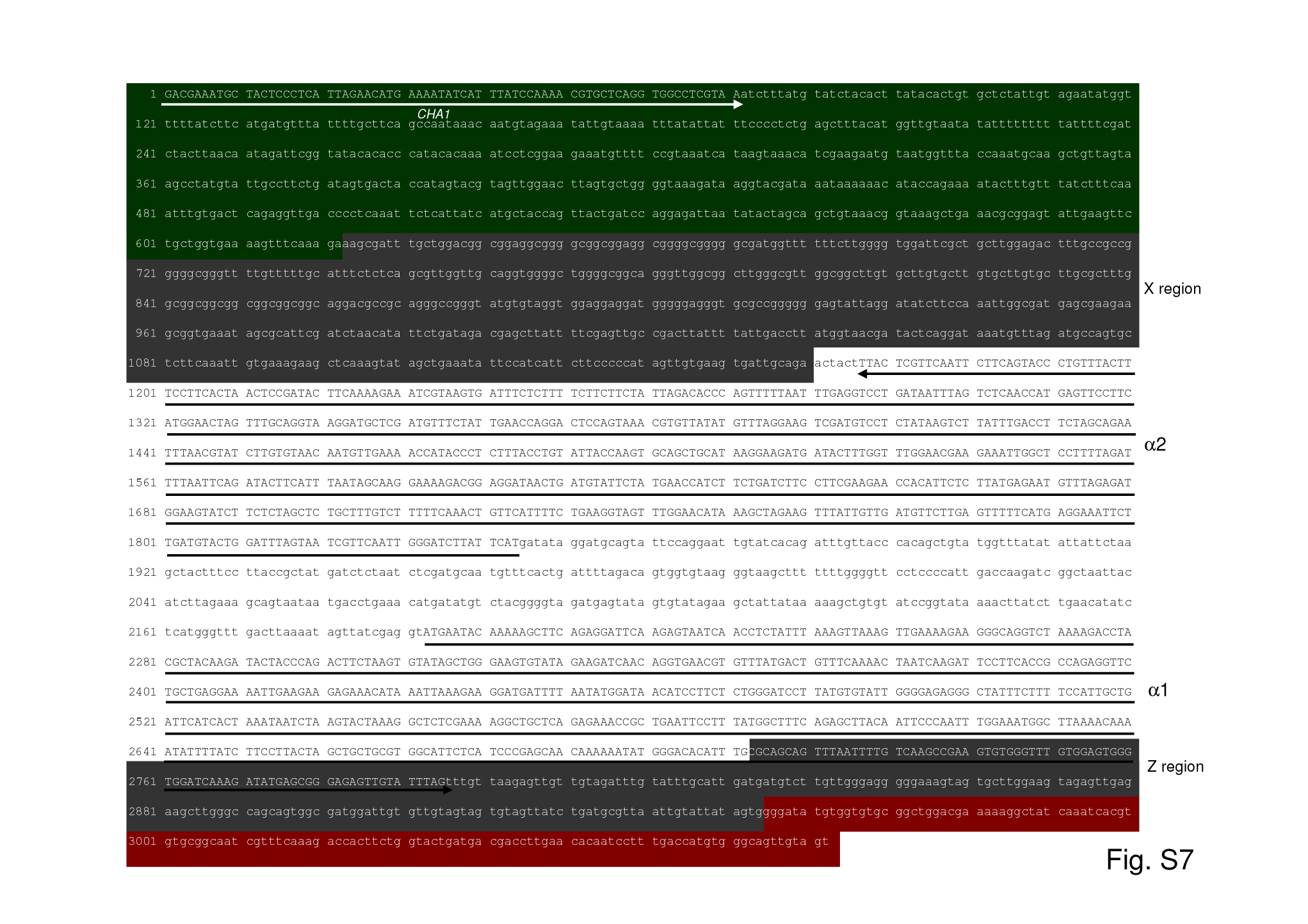

Supplement: Figure S7 — Nucleotide sequence of the HML locus amplified by using primer pair 1′-C′ from NBRC0686 (AB781028). Uppercase lettering indicates putative open reading frame. Arrows indicate gene direction. Coloring indicates gene position in NBRC1130: dark green, left side of HMR; gray, X and Z region; and wine red, right side of HML. (TIF) [file pone.0062121.s007.tif]

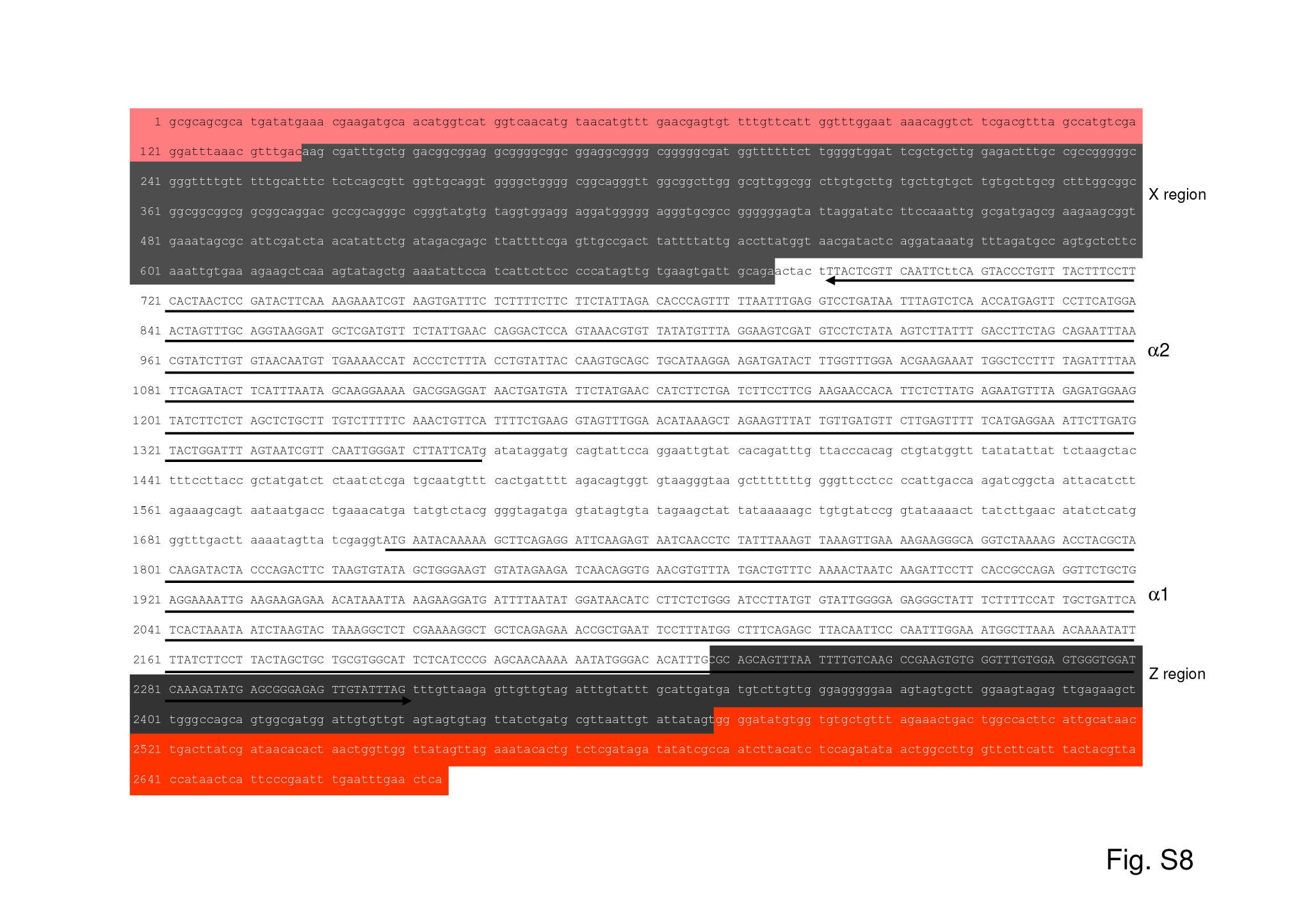

Supplement: Figure S8 — Nucleotide sequence of the MAT locus amplified by using primer pair 2′-A′ from NBRC1130 (AB781020). Uppercase lettering indicates putative open reading frame. Arrows indicate gene direction. Coloring indicates gene position in NBRC1130: pink, left side of MAT; gray, X and Z region; and orange, between MAT and HML. (TIF) [file pone.0062121.s008.tif]

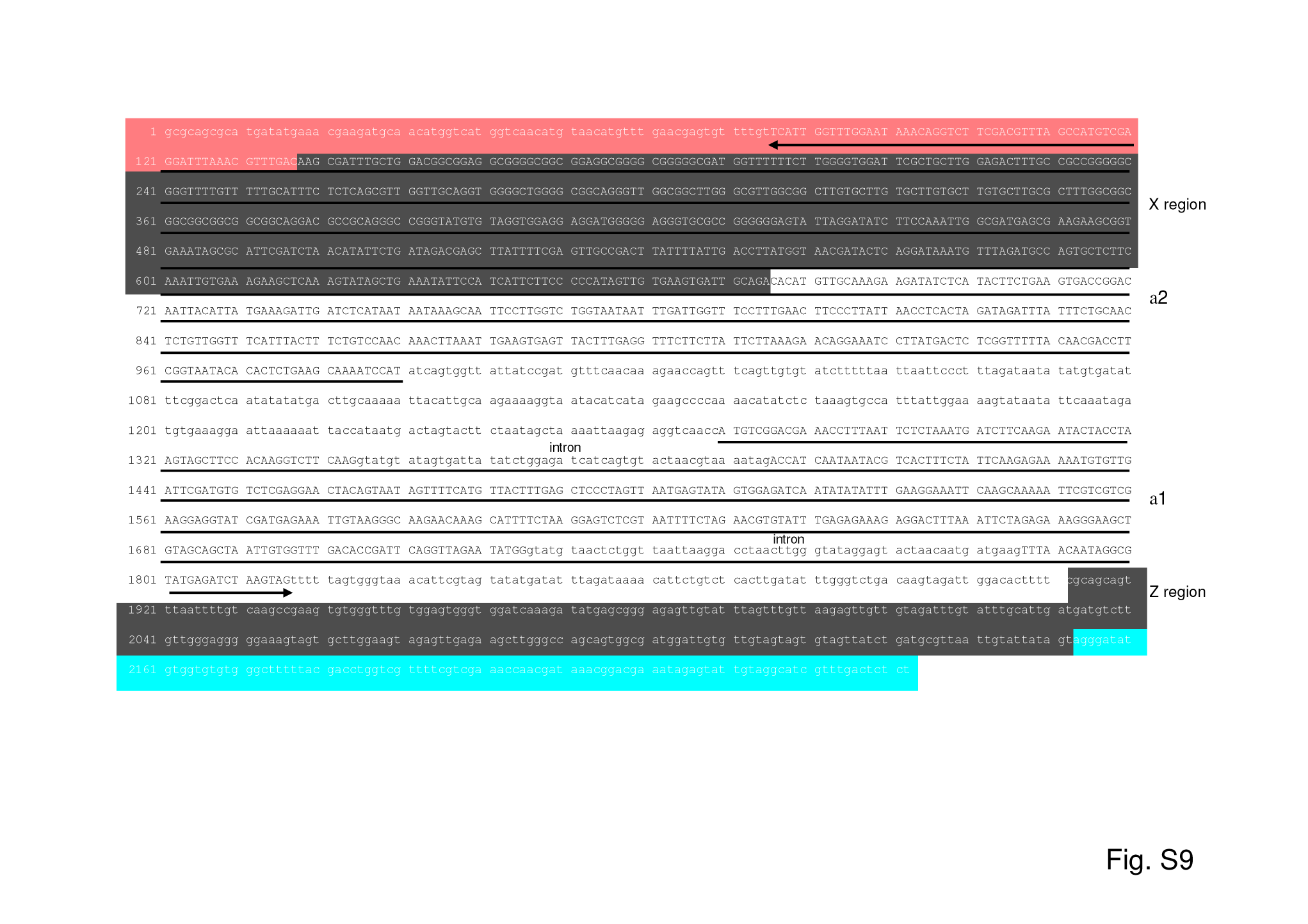

Supplement: Figure S9 — Nucleotide sequence of the putative HMR locus amplified by using primer pair 2′-B′ from DA2 (AB791018). Uppercase lettering indicates putative open reading frame. Arrows indicate gene direction. Coloring indicates gene position in NBRC1130: pink, left side of MAT; gray, X and Z region; and light blue, right side of HMR. (TIF) [file pone.0062121.s009.tif]

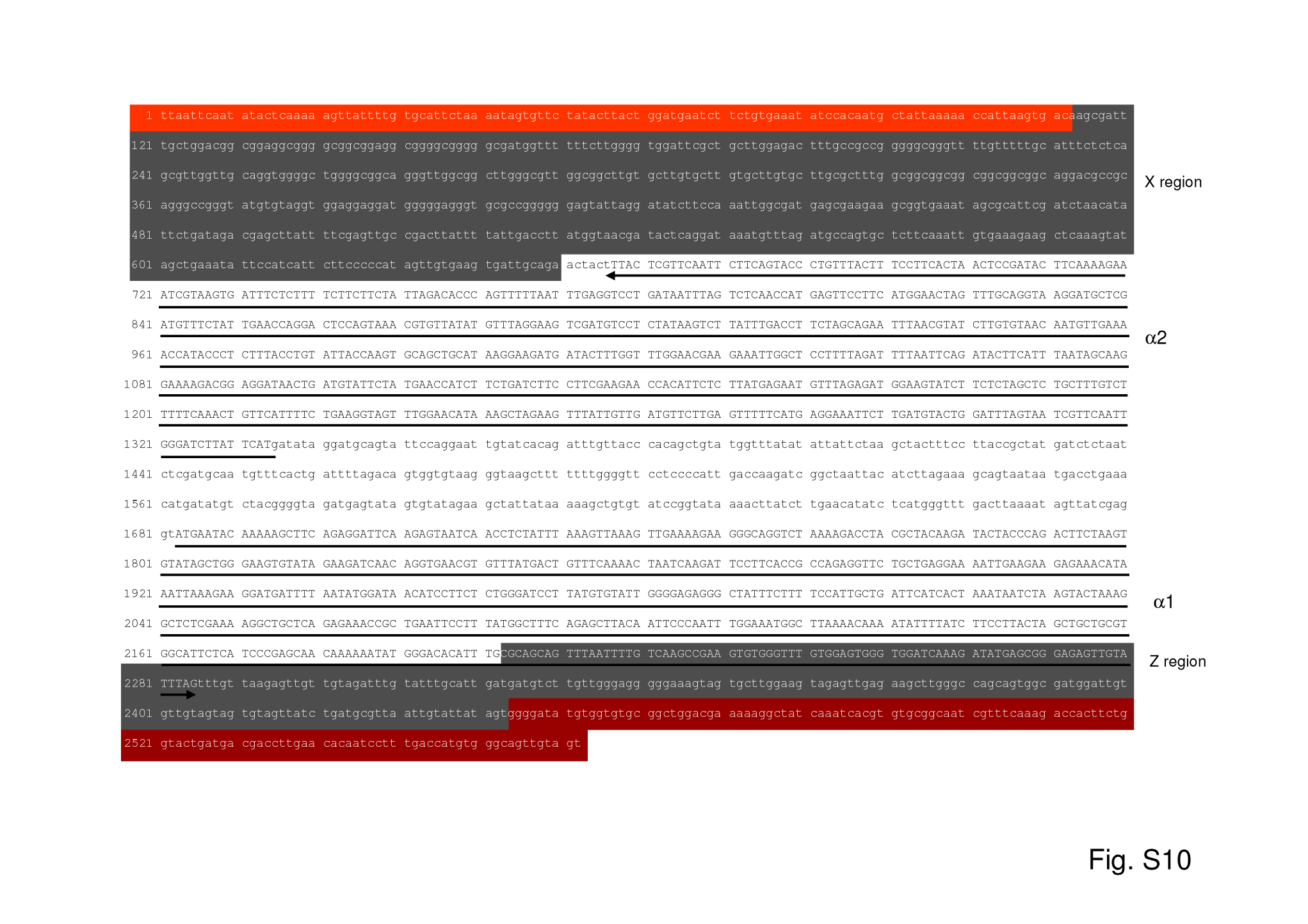

Supplement: Figure S10 — Nucleotide sequence of the putative HML locus amplified by using primer pair 3′-C′ from DA2 (AB781019). Uppercase lettering indicates putative open reading frame. Arrows indicate gene direction. Coloring indicates gene position in NBRC1130: orange, between MAT and HML; gray, X and Z region; and wine red, right side of HML. (TIF) [file pone.0062121.s010.tif]
